# Mitochondrial dysfunction in heart diseases: Potential therapeutic effects of *Panax ginseng*

**DOI:** 10.3389/fphar.2023.1218803

**Published:** 2023-07-20

**Authors:** Xinxin Cao, Fan Yao, Bin Zhang, Xiaobo Sun

**Affiliations:** ^1^ Institute of Medicinal Plant Development, Peking Union Medical College and Chinese Academy of Medical Sciences, Beijing, China; ^2^ Key Laboratory of Bioactive Substances and Resources Utilization of Chinese Herbal Medicine, Ministry of Education, Beijing, China; ^3^ Beijing Key Laboratory of Innovative Drug Discovery of Traditional Chinese Medicine (Natural Medicine) and Translational Medicine, Beijing, China; ^4^ Key Laboratory of Efficacy Evaluation of Chinese Medicine Against Glyeolipid Metabolism Disorder Disease, State Administration of Traditional Chinese Medicine, Beijing, China

**Keywords:** *Panax ginseng*, ginsenosides, mitochondria, heart disease, mechanism

## Abstract

Heart diseases have a high incidence and mortality rate, and seriously affect people’s quality of life. Mitochondria provide energy for the heart to function properly. The process of various heart diseases is closely related to mitochondrial dysfunction. *Panax ginseng* (*P. ginseng*), as a traditional Chinese medicine, is widely used to treat various cardiovascular diseases. Many studies have confirmed that *P. ginseng* and ginsenosides can regulate and improve mitochondrial dysfunction. Therefore, the role of mitochondria in various heart diseases and the protective effect of *P. ginseng* on heart diseases by regulating mitochondrial function were reviewed in this paper, aiming to gain new understanding of the mechanisms, and promote the clinical application of *P. ginseng*.

## 1 Introduction

Heart diseases are one of the world’s top public health concerns. In 2017, approximately 17.8 million people died of cardiovascular disease globally, representing 330 million years of life lost and another 35.6 million years of disabled life (Mensah et al., 2019) According to the Global Burden of Disease Study 2019 (GBD 2019), an estimated 523.2 million people suffer from cardiovascular diseases, including 194.2 million people with ischemic heart disease (Brodmann et al., 2020). It is reported that 290 million people suffer from cardiovascular diseases in China, of which hypertension is the most common, followed by coronary heart disease, heart failure, rheumatic heart disease and congenital heart disease. The number of sudden cardiac death in China exceeds 500,000 every year, ranking first in the world. (Wu, 2022). There are many risk factors for heart diseases, including a family history of heart diseases, obesity, high cholesterol, diabetes, lack of exercise and so on (Fuster, 2014). Heart diseases involves various regulatory mechanisms, including mitochondrial dysfunction, oxidative stress response, myocardial fibrosis and apoptosis (Chen et al., 2023). Therefore, it is of considerable clinical value to understand the pathogenesis of heart diseases and search for therapeutic drugs.

*Panax ginseng* C.A.Mey (*Panax ginseng*) is widely used as a traditional herb in Southeast Asian countries and is gaining popularity worldwide due to its medicinal properties (Lee et al., 2020). The medicinal properties of *P. ginseng* are mainly contributed by its chemical components, such as saponins/ginsenosides, polysaccharides, phenolics, volatile oils, alkaloids, proteins, etc (Baek et al., 2012). Ginsenosides are considered as the primary active components of *P. ginseng* and more than 200 ginsenosides have been isolated from it (Li et al., 2018a; Lee et al., 2020; Liu et al., 2020; Liu et al., 2021; Ratan et al., 2021). According to their aglycone structures, ginsenosides are divided into three groups: dammaranes, ocotillols, and oleananes. The dammarane triterpenes, to which most ginsenosides belong, can be generally further classified as protopanaxadiol (Rb1, Rb2, Rb3, Rd, and PPD) and protopanaxatriol (Rg1, Re, Rf, Rg2 and PPT) (Angelova and Abramov, 2018). A total of 131 kinds of ginsenosides were detected in 70% methanol extracts of roots and stems of *P. ginseng*, and 19 kinds of ginsenosides including Ra1, Ra2, Rb1 and Rb2 were quantitatively analyzed by 4000 QTRAP triple quadrupole tandem mass spectrometry (HPLC-ESI-MS) (Wang et al., 2016). 64 ginsenosides including Rg1, Re, Rf and Rb1 were isolated from 70% ethanol extract of *P. ginseng* flower by UPLC-Q-TOF/MS (Li et al., 2018b). More than 400 compounds were identified from the methanol extracts of *P. ginseng* cultivated in different growth environments, including 81 new compounds, such as hexanoyl-Rd and decadienoyl-Rh1 (Sun et al., 2023).

**FIGURE 1:**
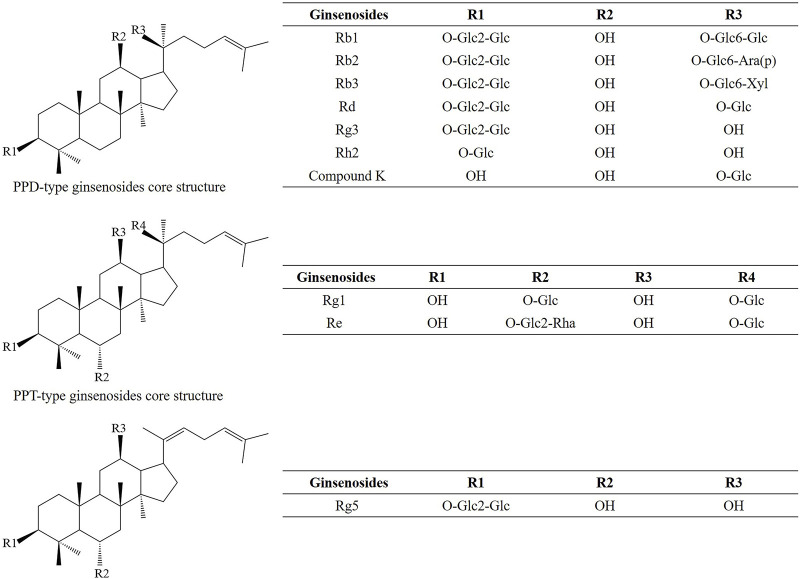
The chemical structures and names of ginsenosides in *Panax ginseng*.

*P. ginseng* extracts obtained by different methods contain a variety of ginsenosides. 12 ginsenosides were isolated from *P. ginseng* root extract by column chromatography with 70% ethanol reflow D101 resin. Namely, Rg1, Re, R0, malonyl Rb1 (mRb1), malonyl Rc (mRc), malonyl Rb2 (mRb2), malonyl Rd (mRd), Rf, Rb1, Rc, Rb2 and Rd (Yi et al., 2010). 11 ginsenosides, including Rg1, Re, Rb1, Rc, Rb2, Rd, Rk3, (20S) Rg3, (20R) Rg3, Rk1, and Rg5, were identified from the ethanol-extracted *P. ginseng* root extract by HPLC (Luo et al., 2015). This paper mainly discusses the protective effects of various ginsenosides (such as ginsenosides Rb1, Rb2, Rb3, Rd, Rg3, Rh2, compound K, Rg1, Re and Rg5; Figure 1) or *P. ginseng* extracts on various heart diseases including improving arrhythmia, reducing myocardial damage, improving mitochondrial dysfunction, inhibiting oxidative stress and apoptosis (Li et al., 2015; Yu et al., 2019; Yuan et al., 2019).

Mitochondria, as a crucial organelle in cell physiology, make up about one-third of the volume of adult cardiomyocytes, and most of the approximately 95% ATP consumed by cardiomyocytes comes from oxidative metabolism in mitochondria (Zhou and Tian, 2018). Therefore, mitochondria are colloquially known as the “energy house” of the cell, as well as the major source of ATP, NADH and NADPH (Disatnik et al., 2015). Mitochondrial dysfunction contributes to the development of cardiovascular and cerebrovascular diseases, cancer, neurodegeneration, and metabolic diseases (Kuida et al., 1998; Zhang et al., 2019; Oh et al., 2019). The overproduction of ROS in mitochondria is also a major cause of cell destruction, which activates the process of programmed cell death or apoptosis (Disatnik et al., 2015).

Numerous studies have verified that *P. ginseng* has a protective effect against various heart diseases by improving mitochondrial dysfunction (Chen et al., 2019; Qi et al., 2020; Wang et al., 2021). In order to better understand how *P. ginseng* and ginsenosides exert pharmacological effects on the basis of mitochondrial dysfunction, this paper reviews the research progress of the protective effect of *P. ginseng* on various heart disease. 1) This paper first reviewed the biological functions of mitochondria, including mitochondria-mediated ROS, mitochondria-mediated apoptosis, mitochondrial dynamics, mitochondrial autophagy and mitochondria-mediated Ca^2+^ homeostasis. 2) The pathophysiological effects of *P. ginseng* on various heart diseases based on mitochondrial dysfunction were reviewed, including diabetic cardiomyopathy, myocardial ischemia/reperfusion injury, cardiac hypertrophy, heart failure and myocardial fibrosis. 3) The regulation and molecular mechanism of *P. ginseng* and different ginsenosides on various heart diseases were reviewed in terms of mitochondrial dysfunction. This study aims to provide new views into the research and application of *P. ginseng* in the area of heart disease.

## 2 Mitochondria Dysfunction in heart diseases

### 2.1 Mitochondrial structure and function

**FIGURE 2:**
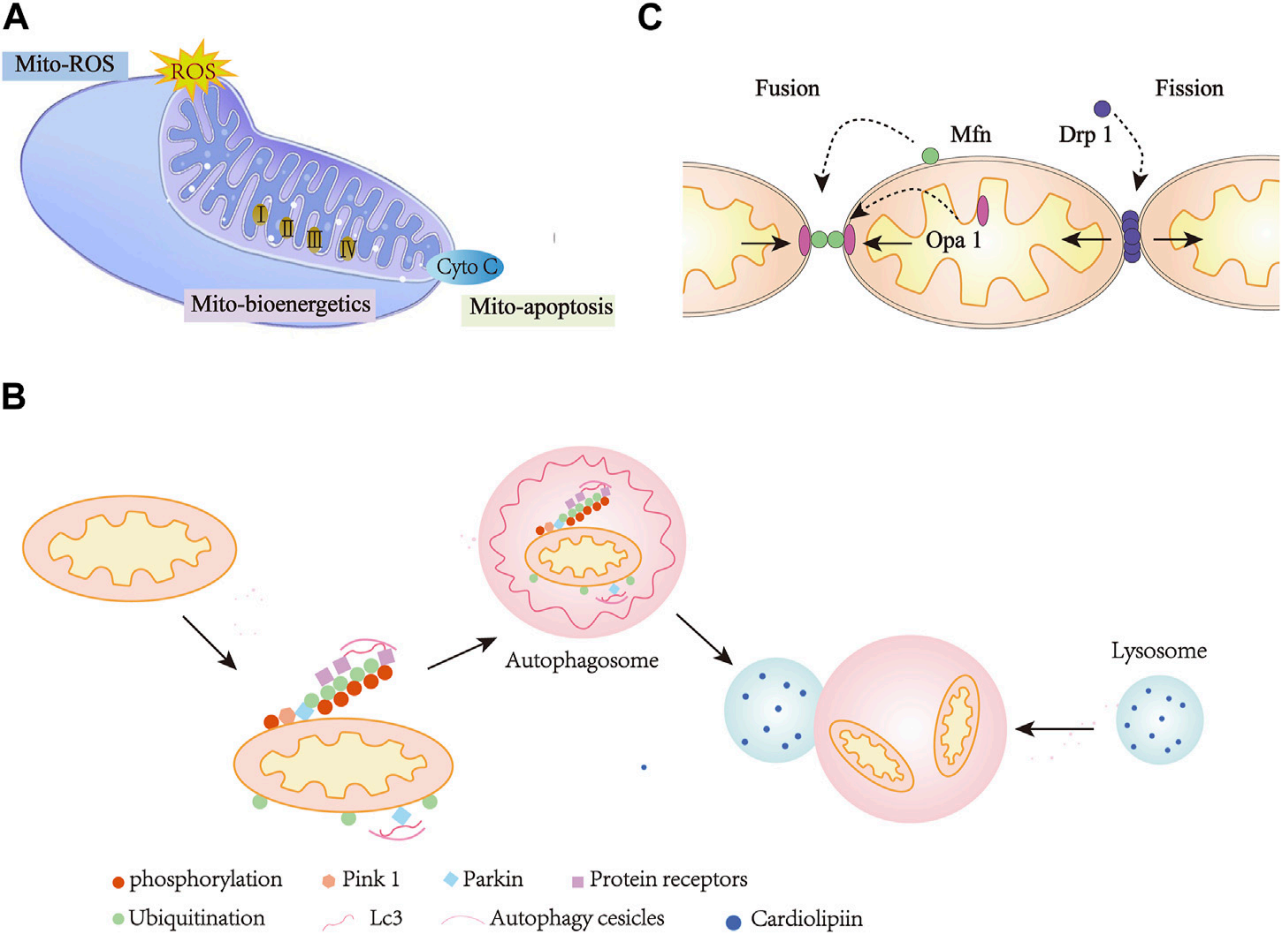
Mitochondria Dysfunction in heart diseases. **(A)**, Mitochondria-mediated ROS, bioenergetics, and apoptosis. **(B)**, Mitochondria mitophagy. **(C)**, Mitochondria dynamics. Mito, Mitochondria; Cyto-C, Cytochrome C.

Mitochondria are organelles found in almost all eukaryotic cells and vary in shape from spherical to rod-shaped to reticular between cell types (Ding et al., 2021). Mitochondria are delineated by outer and inner membrane (Figure 2A). The outer membrane forms an envelope that small molecules can freely penetrate. Because it contains pore-forming proteins, solutes with molecular masses up to several thousand Daltons are also free to permeate, but macromolecules are not (Collins et al., 2002; Pileggi et al., 2021). Morphologically, the inner membrane can be subdivided into inner boundary membrane and the cristae membrane. The inner boundary membrane is closely opposite to the inner membrane and can be regarded as the second envelope structure. The cristae membrane constitutes majority of the inner membrane surface and houses a variety of respiratory complexes containing electron transport chains. In most cases, the cristae form extended sheets, but it may also form tubules or perforated sheets (Frey et al., 2002). The intermembrane space between the inner and outer membranes contains proteins that determine the structure, organization, and folding of mitochondrial proteins (Vogel et al., 2006).

### 2.2 Mitochondria-mediated ROS

The primary function of mitochondria is to generate energy. To ensure cell survival and homeostasis, mitochondria maintain cellular metabolism and high energy supply in the form of ATP through oxidative phosphorylation (OXPHOS) (Zhou and Tian, 2018). Oxidative phosphorylation occurs within the mitochondrial inner membrane, where four large multi-subunit enzyme complexes comprise the electron transport chain, namely, NADH (Figure 2A): ubiquitin oxidoreductase (complex I), succinate dehydrogenase (complex II), coenzyme Q: cytochrome c reductase (complex III), and cytochrome c oxidase (complex IV) (Ashrafi and Schwarz, 2013). In a series of redox reactions, electrons pass through complex I and eventually to complex IV. Using the energy provided in these reactions, ATP synthases can convert ADP into ATP (Newmeyer and Ferguson-Miller, 2003; Ruprecht et al., 2019). The maintenance of mitochondrial structure and integrity is essential for human health. This is especially evident in heart cells that require high energy levels. Mitochondria make up about 30% of the total volume of heart cells and maintain the mechanical function of the heart by producing a surprising amount of ATP through oxidative phosphorylation every day (Hall et al., 2014; Vasquez-Trincado et al., 2016).

Mitochondrial oxidative metabolism is not limited to the production of 6–30 kg ATP per day (Allard et al., 1994; Lopaschuk et al., 2021), but also regulate cellular signaling by modulating redox status, which is also a major source of ROS (Figure 2A). ROS can trigger oxidative stress, which affects cell survival and death (Bhatti et al., 2017; Kiyuna et al., 2018). ROS refers to all active chemicals derived from oxygen, including free radical (e.g., O_2_•^-^, •OH), as well as non-free radicals (e.g., H_2_O_2_) (Yardeni et al., 2019). Free radical superoxide is produced by the reaction of excess electrons with molecular oxygen and is characterized by an initially very active reactive oxygen species. When extra electrons are acquired, it leads to the formation of other forms of ROS, such as •OH and H_2_O_2_ (Bugger and Pfeil, 2020). One of the main sources of ROS in eukaryotic cells is produced by mitochondrial electron transport. *In vitro* experiments have proven that electron leakage from the respiratory chain complex I, II and III leads to the formation of ROS in the mitochondria (Nolfi-Donegan et al., 2020; Kowalcayk et al., 2021). *In vivo* studies have shown that ROS production occurs primarily in the mitochondrial matrix of complex I or intermembrane space of complex III (Goncalves et al., 2015; Newmeyer and Ferguson-Miller, 2003). Eleven sites in the electron transport chain (ETC) and mitochondrial substrate metabolism have been identified as producing ROS. Some studies have suggested that mitochondrial reverse electron transport (RET) is another source of superoxide production (Bugger and Pfeil, 2020). Superoxide production occurs during leakage of electrons, which are transferred from complex II to complex I via ubiquinon reducing NAD^+^ to NADH. Complex I produced ROS as electrons cycle forward or backward (Kowalcayk et al., 2021). In addition, ROS can also be produced by monoamine oxidase and mitochondria localized NADPH oxidase 4 (Kuroda et al., 2010; Kaludercic et al., 2013).

Mitochondrial ROS is critical in physiology and pathology (Angelova and Abramov, 2018). Mitochondria ROS, a byproduct of normal aerobic metabolism, not only regulates signaling within mitochondria, but also regulates signaling cascades outside mitochondria (Bartosz, 2009). For example, ROS can induce the sensitivity of insulin receptors to autophosphorylation and activates G-protein-coupled receptors, growth factors, cytokines, and MAPKs. Various transcription factors, such as NF-κB, Nrf2, p53, HIF-1α, and calcium-treated proteins, such as L-type Ca^2+^ channels, calmodulin, and SERCA2a are also regulated by ROS (Moris et al., 2017). ROS are associated with cell damage, necrosis and cell apoptosis due to their direct oxidizing effects on proteins, lipids and DNA (Ray et al., 2012). It is worth noting that ROS is involved in cell signal transduction as a mediator and regulator of vascular function. The imbalance between ROS and antioxidants under pathophysiological conditions plays an important role in endothelial dysfunction and various cardiovascular diseases (Kim et al., 2016).

### 2.3 Mitochondria-mediated apoptosis

The roles of mitochondria are more than just life-sustaining, they are also actively involved in cell death (Zhou and Tian, 2018). Mitochondrial dysfunction caused by DNA damage and other factors leads to apoptotic cell death (Figure 2A). Mitochondrial fragmentation during apoptosis is considered to be an irreversible signal in the death cascade (Green and Kroemer, 2004). Various diseases associated with heart failure, neurodegeneration, autoimmune and viral infections are mediated by mitochondria-mediated apoptosis (Schneider et al., 2019; Faccenda and Campanella, 2020; Baburina et al., 2021; Steudler et al., 2022).

Programmed cell death or apoptosis is a cellular self-destruction mechanism involving multiple biological events. There are a variety of cellular pathways causing apoptosis, among which the endogenous and exogenous pathways are more specific (Jeong and Seol, 2008). Endogenous pathways are controlled by members of the Bcl-2 family that include pro-apoptotic and anti-apoptotic proteins. Exogenous pathways include cell surface tumor necrosis factor (TNF)-associated receptor families such as TNF receptors, CD95/Fas, and TRAIL death receptors. The process of apoptosis is carried out by the cysteine protease family. These proteases, which are called caspases, specifically lyse their substrates on aspartic acid residues. Caspases are activated by exogenous and/or endogenous pathways (Adams, 2003).

The effect of mitochondria in cardiac apoptosis has been well established (Baines, 2009). Under the conditions of impaired electron transfer chain activity, increased ATP depletion and oxidative stress, dysfunctional mitochondria can initiate the internal mechanism of cardiomyocyte apoptosis (Chistiakov et al., 2018). Mitochondrial outer membrane integrity during apoptosis is connected with members of the Bcl-2 protein family (Harris and Thompson, 2000; Adams and Cory, 2001; Kuwana et al., 2005). Bax and Bak produce cell death via mitochondrial outer membrane permeabilization, resulting in the arrival of small pro-apoptotic molecules from the mitochondrial intermembrane space into the cytoplasm, for example, endonuclease G, cytochrome c, and apoptosis-inducing factors, which subsequently trigger caspase-dependent apoptosis pathways (Jeong and Seol, 2008). The release of cytochrome c further activates caspase-3 and apoptotic protease activator 1, causing nuclear DNA fragmentation and cell death (Palaniyandi et al., 2010). Additionally, dysfunction of mitochondria can lead to endoplasmic reticulum (ER) stress, which subsequently activates calpain whose activation markedly regulates the expression of caspase family (Zuo et al., 2018; Thompson et al., 2020). For example, caspase-12 activation has been widely established to be mediated by calpain (Martinez et al., 2010; An et al., 2019). During cardiac ischemia, a mitochondrial serine protease, such as high temperature requirement A2, is also released from the mitochondria to the cytoplasm, where it is involved in caspase activation and promotes apoptosis (Chistiakov et al., 2018). Mitochondrial dysfunction associated with cardiomyopathy are reversed by targeting apoptotic factors, enzymes and kinases. For example, the increase of Bcl-2 can act an anti-apoptotic role by regulating Ca^2+^ concentration, and possess a protective effect on myocardial injury (Chen et al., 2001). In short, mitochondrial dysfunction plays a crucial role in cardiomyocyte apoptosis.

### 2.4 Mitochondrial dynamics

Mitochondria are highly dynamic organelles that regulate their form, distribution and function through fusion and fission cycles, which are known as “mitochondrial dynamics” (Dorn et al., 2015) (Figure 2B). Disruption of mitochondrial quality control leads to defects in mitochondrial function, which is likely associated with many different complex diseases such as cardiovascular and cerebrovascular diseases, diabetes, cataract and myasthenia (Liesa et al., 2009).

The molecular mechanisms that control the mitochondrial fusion process are highly regulated. Mitochondrial fusion involves two processes, in which MFN1 and MFN2 proteins coordinate the fusion of the mitochondrial outer membrane and optic atrophy factor 1 (OPA1) mediates the intimal fusion (Song et al., 2007; Van der bliek et al., 2013; Disatnik et al., 2015). Mitochondrial fission can also be an important component of mitochondrial quality control, which is used to remove impaired mitochondria during oxidative stress and loss of mitochondrial membrane potential (Twig et al., 2008; Boulton and Caino, 2022). Multiple proteins work together to control mitochondrial fission, including fission protein, dynamin-related protein 1 (DRP1) (Fonseca et al., 2019), mitochondrial fission factor (MFF) (Toyama et al., 2016), mitochondrial fission protein 1 (FIS1) (Zhang and Chan, 2007), and the mitochondrial dynamics proteins (MiD49 and MiD51) (Osellame et al., 2016).

As a major fission promoting protein, DRP1 activity is strictly regulated. DRP1 lacks mitochondrial target sequence. Therefore, DRP1 needs to be collected and assembled on the outer membrane by MFF and FIS1 to form a fission complex (Losóna et al., 2012; Yu et al., 2017; Pileggi et al., 2021). Multiple post-translational modifications (PTMs) of DRP1, including phosphorylation, ubiquitination, S-nitrosylation, palmitation, SUMOylation and O-GlcNAcylation, play a critical role in the regulation of mitochondrial dynamics (Jin et al., 2021). For example, phosphorylation of Cdk1/cyclin B kinase increase DRP1 fission activity (Chang and Blackstone, 2007; Taguchi et al., 2007; Song et al., 2021). Fission mitochondrial fragments can be observed with electron microscopy in many different heart diseases, including acute myocardial ischemia/reperfusion (MI/R) injury, myocarditis, stroke, doxorubicin cardiotoxicity, septicaemia related cardiomyopathy, and diabetic cardiomyopathy (DCM) (Rovira-Llopis et al., 2017; Hernandez-Resendiz et al., 2020).

### 2.5 Mitophagy

Mitophagy is one of the main mechanisms of mitochondrial quality control (Dombi et al., 2017) (Figure 2C). In healthy cells, mitophagy is a tightly controlled process (Scherz-Shouval and Elazar, 2007). In damaged cells, mitochondria can be targeted to remove damaged mitochondria through mitophagy (Campos et al., 2016). The autophagy degradation of mitochondria may be due to a variety of causes, such as basal turnover for recycling, damage and starvation induced degradation (Youle and Narendra, 2011). Mitophagy can also be strongly amplified under various pathological stimulation. The core mechanism of autophagy is highly conserved in evolution, and signaling cascades mediate selective autophagic processes (Xie and Klionsky, 2007).

Mitophagy is a special form of autophagy, including putative kinase protein 1 (PINK1)/Parkin-mediated mitophagy, ubiquitin-mediated mitophagy, BNIP3/NIX/FUNDC1 pathway, neuronal mitophagy and mitophagy *in vivo* (Pankiv et al., 2007; Vives-Bauza et al., 2010; Sarraf et al., 2013; Cai and Jeong, 2020; Zhang et al., 2021). Phosphatase and tensin (PTEN)-induced PINK1/Parkin pathway is the most widely characterized mitophagy pathway (Narendra et al., 2010; Yoo and Jung, 2018; Pileggi et al., 2021). Mitophagy is also activated during hypoxia by inducing junction proteins such as FUN14 domain 1 (FUNDC1), B-cell lymphoma 2 kDa interacting protein 3 (Bnip3) and its analog NIX. All three belong to mitochondrial outer membrane proteins (Novak et al., 2010; Chen et al., 2014; Drake et al., 2017).

Mitochondrial fusion, fission, and autophagy jointly maintain mitochondrial and cellular homeostasis (Vásquez-Trincado et al., 2016). It is now accepted that mitochondrial fission precedes autophagy (Ferro et al., 2020). Mitochondrial fission factor Kinetics Associated protein (Drp) 1 interacts with the mitophagy proteins Parkin and BNIP3 (Lee et al., 2011). Under hypoxia conditions, because FUNDC1 is recruited in the mitochondria-associated ER network, the interaction between FUNDC1 and the ER protein calnexin is further strengthened, leading to the recruitment of Drp1, which allows mitochondrial fission and mitophagy (Wu et al., 2016).

### 2.6 Mitochondria-mediated Ca^2+^ homeostasis

Mitochondrial homeostasis is the mechanism that maintains the integrity and function of mitochondria, and mitochondrial Ca2+ homeostasis occupies a prominent position (Zhang et al., 2022). The homeostasis of Ca2+ concentration is controlled by organelles, for example, mitochondria, endoplasmic reticulum and extracellular matrix (Berridge et al., 2000). Mitochondrial Ca2+ homeostasis exerts a suite of key roles in regulating energy metabolism, oxygen free radical production, death mechanism, autophagy and other cellular physiological and pathological processes (Deceypere et al., 2011; Dietl and Maack, 2017). Therefore, the process of many diseases, such as cardiovascular and cerebrovascular diseases, are closely related to mitochondrial Ca2+ homeostasis (Li and Wang, 2014; Qin et al., 2019; Wu et al., 2019).

Mitochondria produce energy through oxidative phosphorylation, this process that depends on Ca2+ concentration (Viola and Hool, 2014). Mitochondrial Ca2+ produces ATP by activating TCA cyclase and ATP synthase. Moreover, there is a positive feedback regulatory relationship between Ca2+ release and ATP production under agonist-activated cellular conditions (Nichols et al., 1994; Maes et al., 2000; Betzenhauser et al., 2008; Baughman et al., 2011). Ca2+ can also promote ROS production by stimulating the TCA cycle and oxidative phosphorylation (Brookes et al., 2004; Modesti et al., 2021). In addition, Ca2+ has a similar effect by improving respiratory rate and decreasing substrate concentration (Alderton et al., 2001). Just as Ca2+ is essential for the production of ROS, ROS also plays an indispensable role in regulating Ca2+ signaling pathways (Peng et al., 2022). ROS oxidizes and regulates ryanodine receptor (RyR), plasma membrane Ca2+-ATPase, inositol 1,4, 5-triphosphate receptor (IP3R) channels, and other Ca2+ transporters (Aon et al., 2010).

Mitochondrial Ca2+ overload increases the risk of cell death (Rizzuto et al., 2008). The inner membrane of mitochondria is impervious to water under physiological conditions (Kwong and Molkentin, 2014). Under pathological conditions, mitochondrial Ca2+ overload resulted in the opening of permeability transition pore (mPTP). This leading to the release of ions and metabolites, production of ROS, cessation of oxidative phosphorylation, followed by ATP hydrolysis, loss of matrix solutes, and mitochondrial decomposition (Rasola et al., 2010). Apoptosis-inducing factors and cytochrome c are released from the membrane gap. These apoptotic factors will activate caspase apoptosis-related proteins and guide cells into the apoptotic stage. Therefore, mitochondrial Ca2+ overload is considered to be one of the pro-apoptotic pathways (Kwong and Reed, 2000). Mitochondrial Ca2+ is considered to be a potential specific signal regulating mitochondrial autophagy (La Rovere et al., 2016). Mitochondrial autophagy is associated with abnormal regulation of Ca2+ in mitochondria-associated membranes. In addition, interruption of Ca2+ signaling pathway between mitochondria and ER can also induce autophagy (Cárdenas et al., 2011; Puri et al., 2019).

## 3 Protective effect of *Panax ginseng* on myocardial ischemia/reperfusion (MI/R) injury based on mitochondrial dysfunction

Myocardial ischemia/reperfusion (MI/R) injury refers to the injury of myocardial cells secondary to the recovery of blood circulation during the treatment of myocardial infarction (thrombolysis and anticoagulation, etc.), which can lead to further expansion of the infarct area, arrhythmia, heart failure, etc (Heusch, 2020). This is the main reason for the high incidence and mortality of myocardial infarction. The pathological mechanism of MI/R injury is quite complex, mainly involving many aspects such as mitochondrial autophagy, mitochondrial biogenesis, mitochondrial fusion, mitochondrial division, mitochondrial oxidative stress and mitochondrial apoptosis (Tombo et al., 2020). Table 1 summarizes the *in vitro* and *in vivo* studies of *P. ginseng* and ginsenosides in MI/R injury so far (Table 1).

**TABLE 1:** *In vitro* and vivo in studies of *Panax ginseng* and ginsenosides in the application of myocardial ischemia/reperfusion injuries.

Compounds	Dosage	Model	Mechanism	Ref
**mitochondria-mediated oxidative stress**				
Rb1	40 mg/kg	SD rats	Oxidative stress	Xia et al. (2011)
Rb1	12.5 μg/mL	H9c2 cells	Oxidative stress, apoptosis	Ai et al. (2015)
Rb2	10, 20 mg/kg	Wistar rats	Oxidative stress, inflammatory	Xue et al. (2020)
Rb3	5, 10, 20 mg/kg	SD rats	Oxidative stress, microcirculatory	Shi et al. (2011)
Rb3	2, 5, 8 μm, 50 mg/kg	H9c2, SD rats	Oxidative stress	Sun et al. (2019)
Rd	50 mg/kg	SD rats	Oxidative stress	Zeng et al. (2015)
Rg1	10, 20, 40, 60 μM	H9c2 cells	Oxidative stress	Li et al. (2017a)
TGS	100, 200 mg/kg	guinea pig	Oxidative stress	Aravinthan et al. (2015)
TGS	20 mg/kg	SD rats	Oxidative stress	Kim and Lee. (2010)
GSE	10 mg/mL	SD rats	Oxidative stress	Maffer Facino et al. (1999)
RGE	250, 500 mg/kg	guinea pigs	Oxidative stress	Lim et al. (2013b)
**mitochondria-mediated apoptosis**				
Rb1	100 μM	H9c2 cells	Apoptosis	Fan et al. (2020)
Rb1	100 μm	H9c2 cells	Apoptosis	Zhang et al. (2019c)
Rb1	2.5, 5, 10, 20, 40 𝜇M	NRCMs	Apoptosis	Yan et al. (2014)
Rb1	6.25, 25, 100 μmol/L	SD rats	Apoptosis, mPTP opening	Li et al. (2015)
Rb l	20 mg/kg	Wistar rats	Apoptosis	Chen et al. (2002)
Rb1	20 mg/kg	Wistar rats	Apoptosis	Guan et al. (2002)
Rb l	20, 40, 80 mg/kg/d	SD rats	Apoptosis	Li et al. (2020)
Rb1	2.5, 5, 7.5 mg/kg	SD rats	Apoptosis	Cui et al. (2017)
Rb1	40 mg/kg/d	SD rats	Apoptosis	Wang et al. (2008)
Rb1	40 mg/kg	SD rats	Apoptosis	Wu et al. (2011)
Rb1	40 mg/kg	SD rats	Apoptosis	Li et al. (2015)
Rb 3	2, 5 μmol/L	H9c2 cells	Apoptosis, inflammation	Ma et al. (2014)
Rb3	40 μM	H9c2 cells	Apoptosis	Chen et al. (2019)
Rd	50 mg/kg	SD rats	Apoptosis	Wang et al. (2013a)
Re	20, 40, 80 mg/kg	Wistar rats	Apoptosis	Liu et al. (2002)
Rg1	5 mg/kg/h	SD rats	Apoptosis, energy metabolism	Li et al. (2018c)
Rg3	5, 20 mg/kg	SD rats	Apoptosis, inflammation	Zhang et al. (2016)
Rg3	10 mM, 60 mg/kg	NRCMs, SD rats	Apoptosis	Wang et al. (2015)
CK	2, 4, 8 μm	H9c2 cells	Apoptosis	Li et al. (2018b)
CK	10 mg/kg	C57BL/6 mice	Apoptosis	Tsutsumi et al. (2011)
TGS	50 𝜇g/mL, 50 mg/L	HAECs, SD rats	Apoptosis	Yi et al. (2010)
GSE	80 mg/kg/d	SD rats	Apoptosis	Luo et al. (2015)
GSE	80 mg/kg/d	SD rats	Apoptosis	Luo et al. (2013)
GSE	20, 40, 80 mg/kg/d	SD rats	Apoptosis	Zhou et al. (2011)
Ginsenosides	2 μmol/L	H9c2 cells	Apoptosis, inflammation	Feng et al. (2018)
**Other**				
Rb1	50 mg/kg/d	ICR mice	Fatty acid, oxidation	Li et al. (2017b)
Rg1	12.5 μM	H9c2 cells	mitochondrial dynamics	Dong et al. (2016)
Rg1	100 𝜇mol/L	H9c2 cells	Autophagy	Zhang et al. (2012)
Rg1	12.5 μmol/L	H9c2 cells	Nutritional stress	Xu et al. (2019)
Re	30, 100 μm	SD rats	Hemodynamics functions	Lim et al. (2013a)
Re	100 μM	HL-1 cells	Ubiquitination	Sun et al. (2020b)
Rg5	10 𝜇M, 50 mg/kg	NRVMs, ICR	Mitochondrial morphological, functional integrity	Yang et al. (2017)
TGS	50 mg/L	SD rats	Energy, metabolism	Wang et al. (2012)

TGS: total ginsenosides; GSE: ginseng extract; RGE, red ginseng; CK, Ginsenoside compound K.

### 3.1 *Panax ginseng* protects MI/R injury through mitochondria-mediated oxidative stress

**FIGURE 3:**
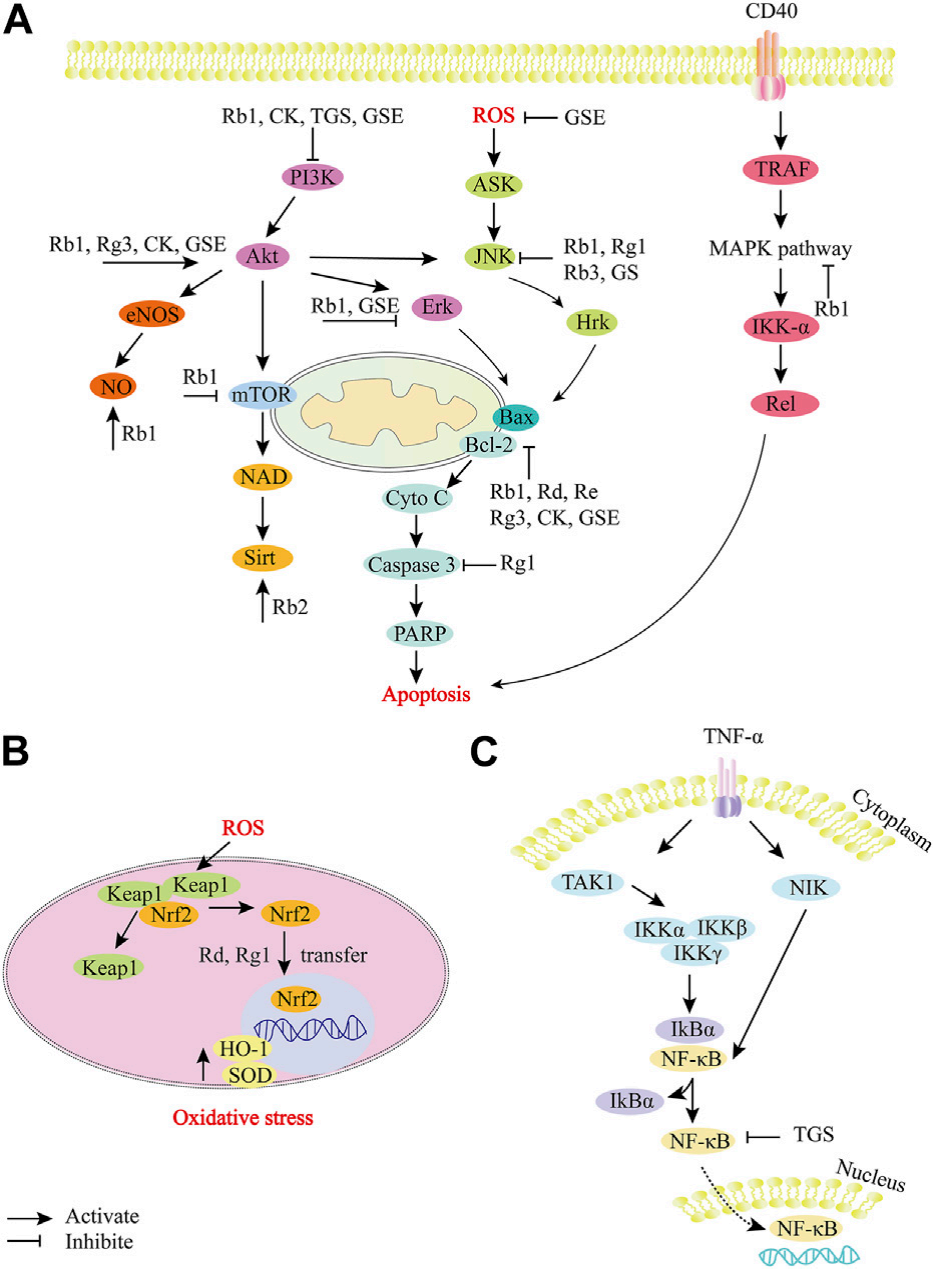
Role and molecular mechanism of *P. ginseng* and ginsenosides in the signaling pathway of myocardial ischemia/reperfusion injury. **(A)**, *P. ginseng* and ginsenosides regulate apoptosis-related pathways. **(B)**, *P. ginseng* and ginsenosides regulate oxidative stress-related pathways. **(C)**, *P. ginseng* and ginsenosides regulate NF-κB related pathways. During the development of myocardial ischemia/reperfusion injury, a variety of molecular mechanisms are involved in the regulation, including apoptotic factors (Bcl, Bax, caspase family), oxidative stress-related substances (ROS, NO), etc. Ginsenosides (Rb1, Rb2, Rg3, Rd, Rg1, Re), CK, TGS and GSE can effectively target different molecular mechanisms, such as inhibiting ROS expression, JNK signaling pathway, caspase-dependent apoptosis pathway, MAPK signaling pathway, etc. TGS: total ginsenosides; GSE: ginseng extract; CK, Ginsenoside compound K; GS: ginsenosides; ERK, extracellular signal-regulated kinase; PI3K, Phosphatidylinositol 3-hydroxykinase; JNK, Jun Amino-terminal kinase.

**Ginsenoside Rb1:** Rb1 pretreatment obviously reduced infarct size and MDA level after local myocardial ischemia reperfusion, and enhanced eNOS expression, NO concentration and SOD activity (Figure 3A; Xia et al., 2011). NO is an important vascular protective molecule, which is related to myocardial cell function, neutrophil activation and free radical production (Jones et al., 2003). Therefore, Rb1 can reduce MI/R injury by reducing oxidative stress (Xia et al., 2011). MI/R injury is a major factor affecting the prevalence of acute myocardial infarction (Yellon et al., 2007). Ai et al. (2015) used hypoxia/reoxygenation (H/R) model to study whether Rb1 has a protective effect on acute myocardial infarction. H/R-induced injury increased ROS concentration in H9c2 cardiomyocytes and MDA levels, a marker of oxidative stress. Rb1 preconditioning effectively reversed the above injury, suggesting that Rb1 can play a cardiac protective role by alleviating H/R -induced oxidative stress in H9c2 cardiomyocytes (Ai et al., 2015).

**Ginsenoside Rb2, Rb3:** Similarly, Rb3 treatment can also dramatically decrease MDA level and enhance SOD expression. This suggests that the cardioprotective properties of Rb3 are related to antioxidant activity (Shi et al., 2011). Rb2 has been reported to reduce myocardial superoxide production, mRNA expression levels and activity of IL-1β, IL-6, and TNF-α. In addition, it can upregulate the expression of Sirt1 and downregulate the expression of Ac-p53. These results suggest that ginsenoside Rb2 alleviates MI/R damage in rats by activating Sirt1 to inhibit oxidative stress and inflammation (Figure 3A; Xue et al., 2020).

**Ginsenoside Rd, Rg1 and Rb3:** Nrf2 is a transcription factor associated with oxidative stress in cardiovascular diseases, and is activated in a tissue-specific manner in response to ROS produced by mitochondria or NADPH oxidase (Cominacini et al., 2015). Nrf2 regulates intracellular redox homeostasis by synergistically regulating a series of antioxidant enzymes and proteins (Tsushima et al., 2020). Rd, Rg1 and Rb3 can alleviate MI/R injury by mediating nuclear factor erythroid 2 associated factor 2 (Nrf2) -related pathways. Rd alleviates MI/R injury by activating the Nrf-2/HO-1 signaling pathway. After Rd treatment, the levels of LDH and Cardiac troponin I (cTnI) decreased, while the expressions of Nrf2 and HO-1 increased (Figure 3B; Zeng et al., 2015). The beneficial effect of Rg1 on H9c2 cells was related to the activation of Nrf2/HO-1 and the inhibition of JNK pathway. Rg1 increased the levels of SOD, GSH-Px and GSH in H9c2 cells, resulting in reduced ROS expression and reduced mitochondrial membrane depolarization. Treatment with Rg1 resulted in Nrf2 nuclear translocation and enhanced HO-1 expression, and reversed H/R-enhanced phosphorylated-JNK levels (Figures 3A,B; Li et al., 2017). Rb3 reduces oxidative stress *in vivo* and *in vitro* by activating the PERK/Nrf2/HMOX1 antioxidant signaling pathway. In H9c2 cells, Rb3 preconditioning inhibited ROS accumulation, enhanced T-AOC, and partially saved H/R-induced oxidative stress and cardiomyocyte apoptosis. In MIR/I rats, Rb3 increased total antioxidant levels, induced PERK phosphorylation and nuclear translocation of transcription factor Nrf2, and promoted the expression of antioxidant genes such as HMOX1, thereby reducing the size of myocardial infarction (Sun et al., 2019).

**Ginseng extract:** In addition to the reported ginseng monomers, total ginsenosides (TGS), ginseng extract (GSE) and red ginseng extract (RGE) have also been reported to reduce MI/R injury. TGS protect myocardial cells from MI/R injury by alleviating oxidative stress. TGS normalizes levels of MDA, GSH and nitrite, which are oxidative stress markers. In addition, TGS can significantly inhibit the expression of IL-1β, IL-6 and NF-κB, and enhance the expression of IL-10 in heart tissue (Figure 3C; Aravinthan et al., 2015). TGS pretreatment inhibited the increase of LDH and MDA levels, and inhibited the decrease of GSH levels, suggesting that it could improve MI/R injury by reducing oxidative stress (Kim and Lee, 2010). Maffer Facino et al. explored the effect of GSE on myocardial ischemia injury induced by hyperbaric oxygen. The results confirmed that GSE could significantly reduce MI/R injury and extracardiac endothelial function injury caused by ROS explosion through antioxidant intervention (Maffer Facino et al., 1999). In addition, RGE may also protect against heart damage by improving biochemical and oxidative stress. It significantly inhibited LDH, creatine kinase-MB components and cardiac troponin I, and improved levels of oxidative stress markers (e.g., MDA and GSH) (Lim et al., 2013b).

### 3.2 *Panax ginseng* protects MI/R injury through mitochondria-mediated apoptosis

Mitochondria-mediated apoptosis plays a crucial role in MI/R injury. Many studies have reported that ginsenosides or ginseng extracts relieve MI/R injury through mitochondria-regulated apoptosis pathway. Rb1 was the most commonly reported, followed by ginseng extract, Rb3, Rg3 and CK. The signaling pathways involved mainly include caspase-induced apoptosis pathway, PI3K-Akt signaling pathway, NF-κB signaling pathway, etc (Figure 3).

**Ginsenoside Rb1:** *In vitro* studies confirmed that Rb1 involved in cardiac protection by inhibiting the mitochondrial apoptosis pathway. The cardioprotective effect of Rb1 is associated with inhibition of mPTP opening by stabilizing mitochondrial membrane potential (Li et al., 2016; Zhang et al., 2019). Rb1 also significantly reduced ROS production, restored mitochondrial transmembrane potential, and inhibited caspase family-dependent apoptotic pathways (Figure 3A; Fan et al., 2020; Yan et al., 2014).

It has been confirmed *in vivo* that cardiomyocytes apoptosis in rats was observed by transmission electron microscopy. Compared with the model group, Rb1 significantly inhibited cardiomyocytes apoptosis in MI/R injury rats. The mechanism may be related to scavenging oxygen free radicals and blocking the influx of extracellular calcium. ROS and Ca^2+^, as important messengers of apoptosis signal transduction, can directly activate the caspase cascade and induce apoptosis (Chen et al., 2002). Similarly, Guan et al. (2002) also used transmission electron microscopy to find apoptotic cells in myocardial ischemic area in MI/R injury group, and the apoptotic cells were reduced by 2.5 times after Rb1 treatment (Guan et al., 2002). Transmission electron microscopy has been used not only for the study of Rb1, but also for the study of Re. Liu et al. confirmed cardiomyocyte apoptosis by transmission electron microscopy, *in situ* nick end labeling (TUNEL) method and light microscopy. The mechanism of Re inhibiting cardiomyocyte apoptosis was related to inhibiting the expression of pro-apoptotic gene Bax and increasing the ratio of Bcl-2/Bax (Table 1; Liu et al., 2002).

Numerous *in vivo* studies have shown that Rb1 prevents MI/R-induced cardiomyocytes apoptosis through multiple pathways. The beneficial effect of Rb1 on cardiomyocytes apoptosis induced by MI/R injury is connected with the activation of mTOR signaling pathway. Compared with MI/R group, Rb1 preconditioning improved cardiac function indicators, reduced cleaved caspase-3 expression, and significantly upregulated the mTOR pathway (Figure 3A; Li et al., 2020). Rb1 may prevent MI/R injury by regulating RhoA/ROCK signaling pathways. Activated RhoA/ROCK can aggravate MI/R injury by mediating myocardial apoptosis, inflammatory myocardial remodeling, and myocardial fibrosis. Rb1 preconditioning directly binds to RhoA, inhibits the activation of RhoA/ROCK1 signaling pathway, and restores ATP production during MI/R injury (Cui et al., 2017). Similar to Rb1, the mechanism of Rg1 to MI/R injury is also through binding with RhoA, inhibiting myocardial apoptosis and regulating energy metabolism (Li et al., 2018).

The protective effect of Rb1 on MI/R injury may also be completed by mediating PI3K/Akt signaling pathway. Rb1 pretreatment significantly decreased infarct size and increased Akt phosphorylated expression. The action of Rb1 was cancelled by the PI3K inhibitor wortmannin (Wang et al., 2008; Wu et al., 2011). It has been indicated that Rb1 prevents H/R-induced H9c2 cardiomyocyte apoptosis through PI3K/Akt/Nrf2/HO-1 signaling pathway (Ai et al., 2015). The p38 mitogen activated protein kinase (MAPK) pathway was also participate in the cardioprotective effect of Rb1. Compared with the model group, Rb1 reduced myocardial infarction size, and caspase-3 activity, TNF-α levels, and phosphorylated p38 MAPK levels were also reduced by Rb1 pretreatment (Figure 3A; Li and Ji, 2018).

**Ginsenoside Rb3:** *In vitro* studies have shown that Rb3 inhibits ROS production and mediates the proteins expression associated with the NF-κB signaling pathway in oxygen-glucose deprivation reperfusion (OGD-rep) MI/R models. The protective effect of Rb3 on MI/R damage was realized by inhibiting the NF-κB pathway mediated by JNK (Figure 3A; Ma et al., 2014). Chen et al. (2019) demonstrated that Rb3 treatment upregulates the expression of mitochondrial deacetylase sirtuin 3 (Sirt 3), peroxidation-activating receptor α (PPARα), and key enzymes related to fatty acid β-oxidation. These results suggest that Rb3 can maintain mitochondrial membrane integrity and inhibit cell apoptosis (Chen et al., 2019).

**Ginsenoside Rg3:** After treatment with Rg3, the activity of Bcl-2 was enhanced, while the activities of Bax, caspase-3 and inflammation-related factors (TNF-α and IL-1β) were decreased. This suggests that the cardioprotective effect of Rg3 is related to its anti-apoptotic and anti-inflammatory effects (Zhang et al., 2016). Wang et al. (2015) also demonstrated cardioprotective effects of Rg3 on MI/R-induced apoptosis mediated by the Bcl-2/Bax pathway and Akt/eNOS signaling pathway in hypoxia and reoxygen injury models and MI/R models. Rg3 inhibited the expression of caspase-3 and caspase-9, increased the levels of p-Akt, eNOS and the ratio of Bcl-2/Bax, and reduced the apoptosis of neonatal rat cardiomyocytes (Wang et al., 2015). Similar to the mechanism of action of Rg3, Rd mediates cardiac protection against MI/R-induced apoptosis through a mito-apoptotic pathway (Figure 3A; Wang et al., 2013).

**Ginsenoside compound K (CK):** A novel ginsenoside metabolite, CK, is formed by gut bacteria. Previous studies have confirmed that CK inhibits cardiomyocyte apoptosis mainly by activating the Bcl-2/Bax pathway. In H9c2 cells, CK preconditioning alleviates ROS accumulation, restores mitochondrial membrane potential, and inhibits autophagy regulated apoptosis (Li et al., 2018a). In addition, PI3K-Akt signaling pathway is also participate in the cardioprotective effect of CK on MI/R injury. Compared with the control group, CK significantly increased protein kinase B (Akt) and eNOS activities. The cardioprotective effect of CK was blocked by the PI3K inhibitor wortmannin (Figure 3A; Tsutsumi et al., 2011).

**Ginseng extract (GSE):** Total ginsenosides have been reported to significantly increase coronary perfusion flow in MI/R rats in a dose-dependent manner by activating the PI3K/Akt-eNOS signaling pathway (Yi et al., 2010). Ginseng extract showed similar effects, significantly reducing infarct size, increasing serum NO production, and inhibiting serum creatine kinase and lactate dehydrogenase activities. This may be connected with the activation of GR/ER, PI3K-Akt-eNOS cascade and ERK1/2 signaling pathway (Figure 3A; Zhou et al., 2011). Ginseng extract also showed the above effects in rat model of acute MI/R injury (Luo et al., 2013). Luo et al. (2015) reported that long-term ingestion of ginseng extract can also reduce acute MI/R-induced heart damage in middle-aged and elderly rats by activating Akt/eNOS pathway (Luo et al., 2015). Feng et al. (2018) discussed the protective effects of protopanaxadiol (S/R), protopanaxatriol, Rh2, Rg3, Rh1 and Rg2 on H/R injury of H9c2 cardiomyocytes. The S enantiomers of the six ginsenosides targeted H/R cardiomyocyte apoptosis more effectively than the R enantiomers. Ginsenosides alleviate H/R-induced apoptosis by activating AMPK and inactivating JNK signaling pathways (Feng et al., 2018).

### 3.3 *Panax ginseng* protects MI/R injury through mitochondrial autophagy, mitochondrial dynamics, and other aspects

**Ginsenoside Rg1 (Mitochondrial autophagy):** The pharmacological effect of ginsenosides on myocardial autophagy has also been reported. After treatment with Rg1, the activities of AMPKα and mTOR were reversed, and the expressions of LC3B-2 and Beclin-1 were decreased. Rg1 prevents autophagosome formation due to ATP production and elimination of oxidative stress (Zhang et al., 2012).

**Ginsenoside Rb1 and Rg1 (Mitochondrial dynamics):** Mitochondrial dysfunction is a prominent feature of MI/R injury. *In vitro* and *in vivo* studies have confirmed that Rb1 prevents succinic acid accumulation by countering mitochondrial fatty acid oxidation and improves cell metabolic homeostasis, thereby reducing apoptosis during H/R injury (Li Q. et al., 2017). Ginseng has been reported to protect cardiomyocytes damage by regulating mitochondrial dynamics (fusion/fission). In H/R cardiomyocyte model *in vitro*, Rg1 regulates dysregulation of glutamate dehydrogenase, significantly increases mitochondrial length, reduces the number of mitochondrial fragment cells, ultimately prevents mitochondrial dynamic imbalance after H/R injury. The effect of Rg1 was reversed after MFN2 knockout. These results suggest that Rg1 regulates glutamate dehydrogenase and MFN2 to sustain mitochondrial dynamics and ultimately protect cardiomyocytes (Table 1; Dong et al., 2016).

**Other aspects (Rg1 and Re):** Rg1 inhibits cell death, which is caused by glucose competition, by rescuing ATP levels and mitochondrial membrane potential. *In vivo* studies confirmed that Rg1 increased the expression of aldolase, p-AMPK, and PINK1 in the hearts of hungry mice. Moreover, it may limit nutritional stress-induced H9c2 cell damage by controlling the aldolase/AMP activated protein kinase/PINK1 pathway (Xu et al., 2019). Ginsenoside Re had a significant protective effect on MI/R-induced rat hearts, which was manifested as dramatically preventing the decrease of hemodynamic parameters, improving electrocardiogram abnormalities, and inhibiting the level of inflammation marker TNF-α (Lim et al., 2013a). *In vitro*, H/R-induced myocardial damage was mitigated by Re, which may be related to inhibition of HIF-1α ubiquitination (Sun H. et al., 2020). The cardioprotective effect of Rg5 is regulated by mitochondrial hexokinase-II (HK-II) and dynamin-related protein 1 (Drp1). The mechanism of the cardioprotective effect of Rg5 may be to prevent apoptosis by promoting the mitochondrial binding of HK-II and reducing the recruitment of Drp1 to mitochondria (Table 1; Yang et al., 2017). The cardioprotective mechanism of total ginsenosides is related to the activation of tricarboxylic acid (TCA) circulating protein and improvement of myocardial energy metabolism. Energy metabolism-related proteins (e.g., LDHB and ODP-2) were improved by total ginsenosides treatment in MI/R-damaged heart tissue (Wang et al., 2012).

## 4 Protective effect of *Panax ginseng* on cardiac hypertrophy, heart failure and myocardial fibrosis

**FIGURE 4:**
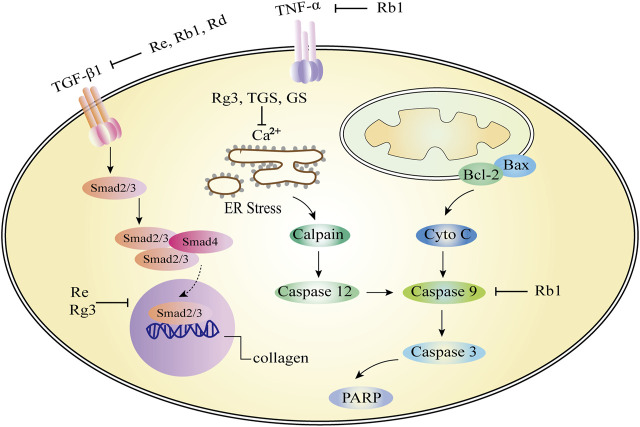
Role and molecular mechanism of *Panax ginseng* and ginsenosides in the signaling pathway of cardiac hypertrophy, heart failure and myocardial fibrosis. A variety of molecular mechanisms are involved in the above regulation, including apoptosis factors (Bcl-2, Bax, caspase family), calcium ions, etc. Ginsenosides (Rb1, Rg3, Re) and TGS can effectively target different molecular mechanisms, such as the regulation of calcium ion concentration, inhibition of caspase-dependent apoptosis pathway, endoplasmic reticulum stress, etc. TNF-α, tumor necrosis factor-α; TGS: Total ginsenosides; GS, ginsenosides; Cyto-C, Cytochrome C.

Myocardial hypertrophy is a risk factor for adverse reactions in patients with cardiovascular diseases. Persistent myocardial hypertrophy is a multifactorial clinical syndrome and a major cause of heart failure (Ho and Wang, 2021). Heart failure is a common and complex clinical syndrome, which is the end-stage manifestation of heart failure in many cardiovascular diseases and the leading cause of death (Elgendy et al., 2019). Inflammation, hypertrophy, apoptosis and fibrosis of cardiomyocytes are multiple factors that lead to heart failure (Figure 4). Table 2 summarizes the *in vitro* and *in vivo* studies of *P. ginseng* and ginsenosides in cardiac hypertrophy, heart failure and myocardial fibrosis so far (Table 2).

**TABLE 2:** *In vivo* and *in vitro* studies of *Panax ginseng* and ginsenosides in the application of cardiac hypertrophy, heart failure and myocardial fibrosis.

Compounds	Dosage	Model	Mechanism	Ref
Rb1	6.25, 25, 100 mg/kg	Cardiac hypertrophy	Inflammation	Wang et al. (2021)
Ginsenosides	0.1, 1.0, 10 μg/mL	Myocytes Cardiomyocyte hypertrophy	NHE-1	Guo et al. (2011)
	100 mg/kg	Heart failure	Calcineurin activity	
Rb1	-, 20 mg/kg	H9c2 cells, ISO-induced apoptosis	Apoptosis	Wang et al. (2013b)
Rg3	25, 50, 100 mg/kg	Heart failure	Ca^2+^	Liu et al. (2021b)
TGS	20 mg/kg	Chronic heart failure	Haemodynamics, Ca^2+^	Li et al. (2009)
Re	5, 20 mg/kg	ISO-induced myocardial fibrosis	Fibrosis, heart failure	Wang et al. (2019)
		Heart Failure		
Rb1	35, 70 mg/kg	Cardiac dysfunction and remodeling	cardiac hypertrophy, myocardial	Zheng et al. (2017)
Rd	50 ug/kg	Cardiac dysfunction and remodeling	cardiac dysfunction	Zhang et al. (2019b)
			fibrosis	
Rg1	50 mg/kg	TAC-induced left ventricular hypertrophy	Fibrosis	Zhang et al. (2013)
Rh2	5 mg/kg	Myocardial fibrosis	Fibrosis	Lo et al. (2017)

TGS: total ginsenosides; GSE: ginseng extract; ISO, isoproterenol.

### 4.1 Protective effect of *Panax ginseng* on cardiac hypertrophy

*P. ginseng* is widely used in China to treat cardiovascular disease. The pharmacological significance of Rb1 on improving myocardial hypertrophy was investigated. *In vivo*, Rb1 reduced angiotensin II induced myocardial hypertrophy, heart inflammation, and systemic inflammation. Mitochondrial function is maintained. In macrophages, Rb1 reduces the phosphorylation of mitogen activated protein kinases (MAPKs) and mitogen activated protein kinases 1/2 (MEK1/2) as well as the production of IL-1β, IL-6, and TNF. The effect of Rb1 in preventing myocardial hypertrophy may be through inhibition of inflammatory mechanisms (Wang et al., 2021). In addition to ginsenoside monomers, anti-myocardial hypertrophy effects of ginsenosides on cardiomyocytes have also been reported. Ginsenosides significantly inhibited NHE-1 activity, intracellular concentrations of Na^2+^ and Ca^2+^, and calcineurin activity in a concentration-dependent manner. The results showed that ginsenosides exerts strong anti-hypertrophy effects by inhibiting NHE-1 dependent calcineurin activation (Figure 4; Guo et al., 2011). At present, there are few reports about the anti-myocardial hypertrophy effect of *P. ginseng*, and it is very potential to explore the effective active ingredients of *P. ginseng* for this effect.

### 4.2 Protective effect of *Panax ginseng* on heart failure and myocardial fibrosis

**Ginsenoside Rg3 and total ginsenosides (Heart failure):** Ca^2+^ homeostasis plays a pivotal role in heart failure. Rg3 reversed isoproterenol-induced Ca^2+^ levels in HL-1 cell hypertrophy models. The cardioprotective effect of Rg3 was cancelled after SUMO1 gene knockout in mice. In addition, mutations at the SERCA2a SUMOylation site block the active role of Rg3 in Ca^2+^ cycling and are associated with ER stress and ROS production (Liu et al., 2021). In the isoproterenol-induced chronic heart failure rat model, the left ventricular peak pressure (LVSP) and the maximum increase rate of left ventricular isovolumic systolic pressure (+dp/dtmax) are significantly increased in the total ginsenosides (TGS) and berberine combined group, while the left ventricular end-diastolic pressure (LVEDP), plasma BNP and cardiomyocyte Ca^2+^ concentrations are significantly decreased. In conclusion, total ginsenosides can improve hemodynamic abnormalities, plasma BNP and calcium overload in cardiomyocytes in chronic heart failure rats (Figure 4; Li et al., 2009).

**Ginsenoside Rb1 and other ginsenosides (Heart failure):** Apoptosis induced by caspase family proteins is a typical mitochondria-dependent pathway. *In vitro* and *in vivo* studies have confirmed that PKA and caspase-9 pathways may be referred to the effect of Rb1 on cardiomyocyte apoptosis (Figure 4; Wang et al., 2013). The heart failure effects of ginsenosides on cardiomyocytes have also been reported. Ginsenosides significantly inhibited NHE-1 activity, which is a key factor in myocardial hypertrophy, myocardial remodeling, and heart failure, suggesting that ginsenosides also contributes to anti-heart failure effects (Guo et al., 2011).

**Ginsenoside Re (Myocardial fibrosis):** According to previous reports, there are few studies on the effect of *P. ginseng* and ginsenosides on myocardial fibrosis, Wang et al. (2019) reported that after 4 weeks of Re treatment, changes in left ventricular systolic blood pressure, left ventricular end-diastolic blood pressure, and positive and negative extremes of the first derivative of left ventricular pressure were improved. These results suggest that Re possess a beneficial effect on protecting myocardial fibrosis and heart failure in rats. Compared with the model group, Re significantly reduced the level of transforming growth factors-β1 (TGF-β1) in serum, and the expression of Smad3 and type I collagen in heart tissue. The results showed that Re can be mediated TGF-β1/Smad3 pathway to improve the isopropyl myocardial fibrosis induced by epinephrine and heart failure (Figure 4; Wang et al., 2019).

**Ginsenoside Rb1 and Rd (Myocardial fibrosis):** Rb1 (70 mg/kg) decreased cardiac hypertrophy and myocardial fibrosis by attenuating the levels of β-myosin heavy chain (β-MHC), atrial natriuretic factor (ANF), periostin, collagen I, Angiotensin II (Ang II), Angiotensin converting enzyme (ACE) and Ang II type 1 (AT1) receptor and reducing left ventricular (LV) weight/heart weight ratio and cardiomyocyte cross-sectional area. Rb1 may inhibit TGF-β1/Smad and ERK signaling pathways, activate Akt pathway, restore cardiac/mitochondrial function, and improve myocardial fibrosis (Figure 4; Zheng et al., 2017). Similar to the mechanism of action of Rb1, Rd can significantly improve the systolic dysfunction, fibrosis, myocardial hypertrophy, inflammation and oxidative stress caused by pressure load in mice. The inhibition of Akt, calcineurin A, ERK1/2 and TGF-β1 signaling pathways in the heart may be the mechanism of Rd to improve cardiac dysfunction and remodeling induced by pressure load (Zhang et al., 2019).

**Ginsenoside Rg1 and Rh2 (Myocardial fibrosis):** In a rat model of TAC-induced left ventricular hypertrophy, Rg1 significantly reduced TAC-induced myocardial fibrosis and left ventricular hypertrophy. The cardioprotective effect of Rg1 was associated with the activation of phosphorylated Akt and the inhibition of p38 MAPK (Table 2; Zhang et al., 2013). Lo et al. (2017) explored the effect of Rh2 on myocardial fibrosis *in vivo* and *in vitro*. *In vivo* studies have shown that Rh2 can significantly reduce the heart weight ratio and myocardial fibrosis in STZ-diabetic rats, and has a protective effect on cardiac function. In cardiomyocytes, Rh2 reduced the levels of fibrotic signaling proteins, including signal sensor and activator of transcription 3 (STAT3), connective tissue growth factor (CCN2), and fibronectin. These effects can be reversed by PPARδ specific siRNA, so Rh2 may improve cardiac function and fibrosis by increasing PPARδ signaling. In conclusion, Rh2 was suitable for development as an alternative treatment for cardiac fibrosis (Lo et al., 2017).

## 5 Protective effect of *Panax ginseng* on diabetic cardiomyopathy (DCM) based on mitochondrial dysfunction

**FIGURE 5:**
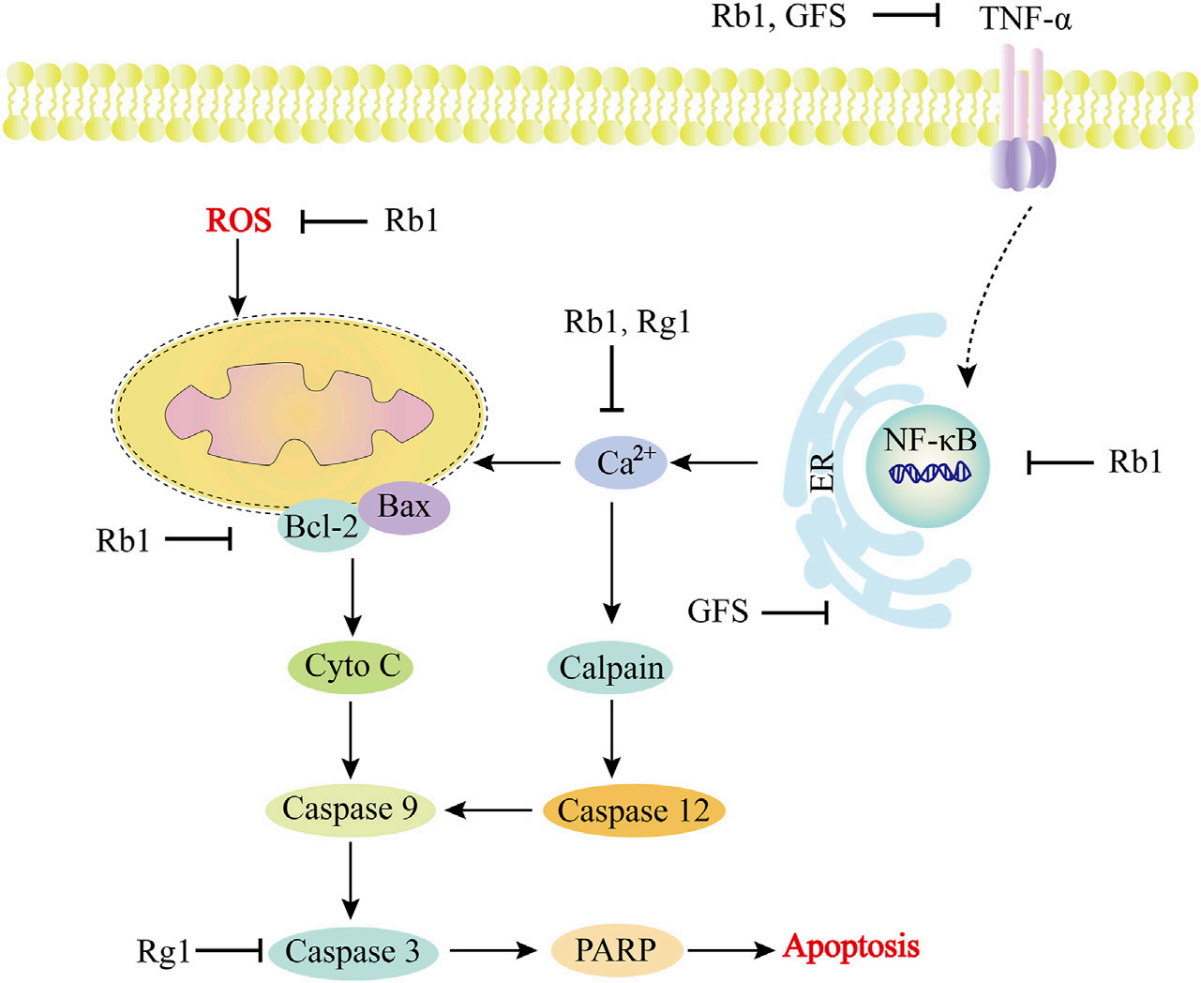
Role and molecular mechanism of *Panax ginseng* and ginsenosides in the signaling pathway of diabetic cardiomyopathy. In the development of diabetic cardiomyopathy, a variety of molecular mechanisms are involved in the regulation, including apoptosis factors (Bcl, Bax, caspase family), oxidative stress-related substances (ROS), calcium ions, etc. The active components of *Panax ginseng* include Rb1 and Rg1, as well as GFS, which can effectively target different molecular mechanisms, such as inhibiting ER stress, regulating Ca^2+^ concentration, inhibiting ROS, and inhibiting caspase family-dependent apoptosis pathways. TNF-α, tumor necrosis factor-α; GFS, Ginseng fruit saponin; Cyto-C, Cytochrome C; PARP, poly ADP-ribose polymerase.

Diabetic cardiomyopathy (DCM) is a disease in which the myocardial functions and structures abnormally in people with diabetes in the absence of other heart disease risk factors, including coronary artery disease, hypertension and valvular disease (Huang et al., 2017). DCM is distinguished by left ventricular hypertrophy, myocardial fibrosis, and impaired left ventricular systolic/diastolic function, ultimately leading to heart failure (Tian et al., 2017). The process of DCM involves diversified mechanisms (Figure 5), including but not limited to oxidative stress, endoplasmic reticulum stress, myocardial apoptosis, mitophagy, impaired calcium processing, myocardial insulin resistance, endothelial dysfunction, mitochondrial dysfunction, etc (Liu et al., 2014; Delbridge et al., 2017). Table 3 summarizes the *in vitro* and *in vivo* studies of *P. ginseng* and ginsenosides in DCM so far (Table 3).

**TABLE 3:** *In vivo* and *in vitro* studies of *Panax ginseng* and ginsenosides in the application of diabetic cardiomyopathy.

Compounds	Dosage	Model	Mechanism	Ref
Rg1	10, 15, 20 mg/kg	STZ-diabetic rat	Oxidative stress, apoptosis	Yu et al. (2015)
Rb1	50 mg/kg	STZ-diabetic rat	Oxidative stress, apoptosis	Qi et al. (2020)
Rb1	25, 50, 100 mg/kg	db/db mice	Oxidative stress, inflammation, apoptosis	Zhang et al. (2022b)
GFS	80 mg/kg	STZ-diabetic rat	Oxidative stress, inflammation	Sun et al. (2020a)
Rb1	10, 20 mg/kg	STZ-diabetic rat	Apoptosis	Zhang et al. (2021a)
Rg1	10, 15, 20 mg/kg	STZ-diabetic rat	Apoptosis	Yu et al. (2016)
GFS	40 mg/kg	STZ-diabetic rat	Apoptosis	Zhao et al. (2014)
Rg1	15, 30, 60 mol/L	myocardial cell	Ca^2+^	Li and Wang. (2014)
Rb1	40 mg/kg	C57BL/6 mice	Ca^2+^	Qin et al. (2019)
Rh2	5 mg/kg	STZ-diabetic rat	Fibrosis	Lo et al. (2017)

GFS: ginseng fruit saponin.

### 5.1 *Panax ginseng* protects DCM through mitochondria-mediated oxidative stress and apoptosis

Oxidative stress is considered to be a key mechanism of DCM induced by diabetes (Hayyan et al., 2016). Mitochondria are the main source of excess superoxide production (Jiang et al., 2019). Excess ROS results in increased oxidative stress, as well as protein, lipid, and DNA damage in cardiomyocytes (Kayama et al., 2015). Apoptosis is one of the crucial mechanisms of myocardial cell injury in DCM. Apoptosis induced by oxidative stress may occur through mitochondrial, death receptor or endoplasmic reticulum stress pathways (Green and Kroemer, 1998; Bäcklund et al., 2004; Voulgari et al., 2010).

**Ginsenoside Rg1:** Previous studies have found that Rg1 protects the myocardium from DCM damage, which may be related to its antioxidant and anti-apoptotic effects (Yu et al., 2015). In diabetic rat model, Rg1 treatment significantly decreased serum and myocardial malondialdehyde levels, and raised superoxide dismutase, catalase and glutathione peroxidase levels compared with normal group. This indicates that Rg1 has a strong ability to reduce the oxidative damage caused by DCM. Rg1 may induce GSH biosynthesis through upregulation of rate-limiting enzymes, thereby enhancing antioxidant activity. TUNEL staining showed that after Rg1 pretreatment for 12 weeks, the apoptosis of rat cardiomyocytes decreased, which may be related to the decrease of caspase-3 level and the increase of Bcl-xL level (Yu et al., 2015). Yu et al. (2016) reported that Rg1 significantly reduced serum cTnI levels, improved cardiac function, and reduced cardiomyocytes damage and apoptosis. The expression of GRP78, CHOP, and caspase-12 proteins decreased, confirming that Rg1 alleviates diabetic myocardial injury by reducing ER stress apoptosis (Table3; Yu et al., 2016). Endoplasmic reticulum and mitochondria are both dynamic organelles (Lepretti et al., 2018). ER and mitochondria cooperate in apoptosis signaling through close contact sites called mitochondria-associated ER membranes (MAMs), which are critical for the regulation of cell homeostasis (Moltedo et al., 2019). However, there is no evidence to confirm the effect of Rg1 or other ginsenosides on MAMs.

**Ginsenoside Rb1:** The mechanism of Rb1 protecting DCM was similar to previous reports (Yan et al., 2014; Fan et al., 2020), that is Rb1 increased mitochondrial biogenesis and reduced the increase of mitochondrial ROS, thus reducing cell apoptosis (Figure 5; Qi et al., 2020). In addition to mitochondria-associated oxidative stress pathways, Rb1 may also prevent DCM by delaying the adipokine pathway and thus activating the antioxidant pathway. Zhang et al. (2022) reported that Rb1 treatment improved serum levels of inflammation-related factors (IL-1β, TNF-α, MCP-1, IL-6, and CRP) and reduced lipid accumulation in diabetic mice after 12 weeks administration. Improvements in cardiac function as well as reductions in oxidative stress, fibrosis, and apoptosis were observed in the heart (Zhang et al., 2022). Hyperglycemia-induced apoptosis of cardiomyocytes is considered to be an important mechanism of DCM (Huynh et al., 2014). Zhang et al. demonstrated that Rb1 has a protective effect on DCM by inhibiting caspase family proteins and NF⁃κB pathway (Zhang et al., 2021), which is similar to the protective effect of Rb1 on MI/R (Figure 3C; Fan et al., 2020; Yan et al., 2014). NF⁃κB is a key factor in triggering inflammatory response and regulating apoptotic response, and its phosphorylation and acetylation are indicative of mitochondria-dependent apoptotic signals (Guan et al., 2018). The expression level of silent information regulator 1 (Sirt1) increased significantly after Rb1 stimulation, suggesting that it can activate Sirt1 (Zhang et al., 2021). Sirt1 is an NAD^+^ dependent protein-modifying enzyme, which can inhibit cardiomyocyte apoptosis and participate in cell metabolism and mitochondrial function (Zhang et al., 2021).

**Ginseng fruit saponin (GFS):** Interestingly, the combination of GFS and total flavonoids of murraya paniculate leaves also contributed to the cardioprotective effect (Figure 5). It obviously enhanced the level of SOD and GSH-Px, and decreased the activities of MDA, suggesting that it could protect DCM by improving oxidative stress damage induced by high sugar. In addition, the activities of IL-6 and TNF-α were also decreased (Sun et al., 2020). Another study showed that GFS treatment significantly increased the level of biochemical indicators (TC, TG, LDH), improved cardiomyocyte abnormalities, and reduced cell apoptosis and caspase-12 protein expression. These results suggest that GFS play a protective effect on DCM by inhibiting endoplasmic reticulum stress-induced apoptosis (Zhao et al., 2014). C/EBP homologous protein (CHOP) is the first key molecule in endoplasmic reticulum stress mediated apoptosis, which regulates the increase of ROS and Ca^2+^ concentrations. CHOP reduced the anti-apoptotic expression of Bcl-2 and Bcl-xl, and enhanced the expression of pro-apoptotic proteins, such as Bax. IRE1 binds to TNF receptor-related factor 2 (TRAF2) to trigger caspase-12, which activates apoptotic factor caspase-3, and finally activates JNK signaling pathway to play the role of apoptosis (Martucciello et al., 2020).

To sum up, to the best of our knowledge, there are few literature on the protective effects of *P. ginseng* and ginsenosides on DCM, and the studies mainly focus on Rb1, Rg1 and GFS. In the future, the beneficial effects of *P. ginseng* and ginsenosides on DCM should be further expanded or explored.

### 5.2 *Panax ginseng* protects against DCM through regulation of mitochondria-mediated Ca^2+^ homeostasis, and improved myocardial fibrosis

**Ginsenoside Rb1 and Rg1:** In addition to protecting DCM through mitochondria-mediated oxidative stress, apoptosis and endoplasmic reticulum stress pathways, Rb1 also plays this role by regulating calcium signaling pathways. Qin et al. found that feeding Rb1 for 8 weeks significantly improved diabetes-induced cardiac dysfunction and abnormal calcium signaling in cardiomyocytes. Rb1 reduces Ca^2+^ leakage caused by overactivated ryanodine receptor 2 (RyR2) and increases Ca^2+^ uptake by sarcoplasmic reticulum Ca^2+^-ATPase 2a (SERCA 2a). In conclusion, Rb1 can not only enhance energy metabolism and regulate calcium processing protein, but also directly inhibit RyR2 activity and regulate calcium signal (Figure 5; Qin et al., 2019). The transfer of Ca^2+^ from the sarcoplasmic reticulum to mitochondria via RyR2 is thought to play a key role in metabolic requirements. The transferred Ca^2+^ enters the mitochondria mainly through the mitochondrial Ca^2+^ single-transporter (MCU) complex (Hamilton et al., 2020). The increased activity of RyR2 disrupted the homeostasis of mito-Ca^2+^ and raised the emission of mito-ROS. This in turn exacerbates RyR2 leaks and contributing to cardiac pathologies (Tow et al., 2022). Rg1 was found to have a protective effect against DCM. Compared with the model group, Rg1 (30 and 60 mol/L) significantly improved cardiomyocyte hypertrophy and inhibited hyperglycemic-induced calcium transient increase, suggesting that the protective effect of Rg1 on hyperglycemic-induced myocardial hypertrophy is related to the inhibition of intracellular calcium overload (Li and Wang, 2014). There are few reports on the beneficial effect of ginsenosides on DCM. Previous studies mainly focused on Rb1 and Rg1, and the protective effects of other ginsenosides on DCM are worthy of further study.

**Ginsenoside Rh2:** Rh2 significantly improved cardiac function and myocardial fibrosis indexes in streptozotocin induced diabetic rats. The effect of Rh2 was reversed by GSK0660 of the oxidase body proliferator-activated receptor (PPARδ), hinting that Rh2 may improve cardiac function and fibrosis by increasing the PPARδ signaling pathway (Lo et al., 2017). The expression of PPARδ was decreased in the heart of diabetic cardiomyopathy rats (Lee et al., 2010). Decreased PPARδ expression in heart cells is associated with ROS production (Chen et al., 2013). Lo et al. also investigated the superoxide levels of H9c2 cells and found that the superoxide levels of H9c2 cells cultured in a high glucose medium were significantly elevated. After treatment with Rh2 and PPARδ, superoxide levels were reduced (Lo et al., 2017). Activation of PPARδ also reduced oxidative stress-induced apoptosis in H9c2 cells (Pesant et al., 2006). Therefore, Rh2 activates PPARδ and inhibits oxidative stress, which may be one of the main mechanisms to reduce myocardial fibrosis in diabetic rats (Table 3).

## 6 Conclusion and future perspectives

*P. ginseng* and ginsenosides have attracted research interest due to their extensive pharmacological actions and medical applications. The protective mechanisms of *P. ginseng* and ginsenosides against various heart diseases mainly focus on restoring mitochondrial membrane potential, regulating Ca^2+^ concentration, and inhibiting caspase-dependent apoptosis pathway. For example, ginsenosides Rb1, Rg1, Rb3, CK and GSE play a protective role in myocardial injury by inhibiting ROS expression and restoring mitochondrial membrane potential, thus maintaining mitochondrial integrity. Rb1, Rg3 and Rd protect against myocardial injury by inhibiting the mitochondrial dependent apoptotic pathway, which is dominated by the caspase-dependent apoptotic pathway. Rb1 and Rg1 also play a role in protecting myocardial injury by regulating mitochondrial dependent Ca^2+^ leakage or Ca^2+^ transient. At present, *P. ginseng* and ginsenosides mainly targets mitochondrial membrane potential and Bcl-2/Bax protein related to mitochondrial apoptosis in alleviating heart disease. Some studies have reported that ginsenoside Rg5 targeted the mitochondrial fission protein DRP1, and Rg1 targeted the mitophagy protein PINK1 to protect against heart injury. Mitophagy protein and fission protein may be new targets of *P. ginseng* and ginsenosides in the treatment of various heart diseases. Previously, formulas containing ginsenoside components have been studied in clinical practice, but the study of ginsenosides to alleviate heart disease is still in the preclinical research stage, and relevant clinical studies are limited. The clinical research of ginsenosides in the field of cardiovascular diseases is the future research direction. This article reviews the related research of *P. ginseng* and ginsenosides in alleviating various heart diseases (such as myocardial ischemia/reperfusion, diabetic cardiomyopathy), in order to provide theoretical basis for clinical research.
